# A Novel In Vitro Wound Healing Assay Using Free-Standing, Ultra-Thin PDMS Membranes

**DOI:** 10.3390/membranes13010022

**Published:** 2022-12-24

**Authors:** Karya Uysal, Ipek Seda Firat, Till Creutz, Inci Cansu Aydin, Gerhard M. Artmann, Nicole Teusch, Aysegül Temiz Artmann

**Affiliations:** 1 Institute for Bioengineering, University of Applied Sciences Aachen/Campus Juelich, Heinrich-Mussmannstr. 1, 52428 Jülich, Germany; 2 Institute for Pharmaceutical Biology and Biotechnology, Heinrich-Heine-University Düsseldorf, Universitätsstr. 1/Geb. 26.23, 40225 Düsseldorf, Germany

**Keywords:** PDMS membranes, membrane functionalization, wound assay, wound healing, mechanical stimulation, CellDrum, ultra-thin membrane, cyclic cell loading, migration rate

## Abstract

Advances in polymer science have significantly increased polymer applications in life sciences. We report the use of free-standing, ultra-thin polydimethylsiloxane (PDMS) membranes, called CellDrum, as cell culture substrates for an in vitro wound model. Dermal fibroblast monolayers from 28- and 88-year-old donors were cultured on CellDrums. By using stainless steel balls, circular cell-free areas were created in the cell layer (wounding). Sinusoidal strain of 1 Hz, 5% strain, was applied to membranes for 30 min in 4 sessions. The gap circumference and closure rate of un-stretched samples (controls) and stretched samples were monitored over 4 days to investigate the effects of donor age and mechanical strain on wound closure. A significant decrease in gap circumference and an increase in gap closure rate were observed in trained samples from younger donors and control samples from older donors. In contrast, a significant decrease in gap closure rate and an increase in wound circumference were observed in the trained samples from older donors. Through these results, we propose the model of a cell monolayer on stretchable CellDrums as a practical tool for wound healing research. The combination of biomechanical cell loading in conjunction with analyses such as gene/protein expression seems promising beyond the scope published here.

## 1. Introduction

Polymer materials are divided into natural and synthetic polymers and have a broad range of applications in biology and medicine. Natural polymeric materials such as silk, cotton, and wool have been in medical use for centuries, whereas synthetic polymers were mostly used in industrial branches, from clothing and household items to electronics and medical applications [1]. Of various synthetic polymers, silicone polydimethylsiloxane (PDMS) is one of the most used polymers in healthcare thanks to its superior bio-properties and biocompatibility [2]. PDMS is chemically inert, stable, optically transparent, gas-permeable, liquid-impermeable, sterilizable, and non-toxic. The material has low surface tension and can resist hot and cold temperatures (−40–150 °C) as well as moisture [1,2,3,4,5,6]. Together with only minor biochemical surface modifications, PDMS membranes are well-suited for applications in medical/biomedical research.

Commercially available PDMS (Sylgard 184 ™) allows fabricating PDMS materials with tunable tensile strength/Young’s modulus by simply changing the curing agent and PDMS base ratio. Generally, a 1:10 ratio is used for mixing the components [7,8]. Curing is either conducted at room temperature over 2 days or in an oven for a couple of hours. In combination with soft lithography, casting, and spin coating, the PDMS materials fabricated are used as coatings for lab-on-a-chip systems, microchips, separation of substances in hemodialysis, water-purification, synthetic biomaterials for stem cell engineering, scaffolds for tissue and organ engineering or even as orthopedic implants [4,5,9,10,11,12,13].

Scientists realized that PDMS could be used to create very thin membranes, especially for utilization in in-vitro studies. This led to the discovery of the patented method of PDMS membrane fabrication by floating on the water surface (FoW) that we have previously reported [13]. With this approach, free-standing, motile PDMS membranes with uniform and tunable membrane thicknesses in the range of 2–10 µm can be produced. In this application, a polycarbonate ring with a diameter of 16 mm is water-tightly enclosed on one side by the ultra-thin PDMS membrane (thickness 4 µm). This arrangement is called CellDrum. To further ensure the successful implementation of CellDrums in biological applications, chemical and biological modifications of the membranes are essential to provide better biocompatibility and cultured cell adhesiveness, mechanical durability, and tunable chemical characteristics [14,15]. This is particularly important as the PDMS surface is highly hydrophobic and hinders work with liquids and proteins; hence is not suitable in its native state for cell survival making surface modifications a necessity [1,4,6,15]. Ultimately, the CellDrum membranes coated, as mentioned before, can be used to apply and measure mechanical tension/stress with the help of devices specifically produced for this system and to test pharmaceuticals and simulate disease models in-vitro [13,16,17,18]. We have previously shown that the CellDrum set-up can be used to quantify the mechanical compliance of self-beating mouse embryonic stem cell-derived cardiomyocytes as a model mimicking septic cardiomyopathy in-vitro [17].

The skin is an elastic and dynamic tissue scaffolded by extracellular matrix (ECM) elements. The mechanical behavior of skin is mediated by ECM structure and fibroblasts’ ability to sense the surrounding biomechanical cues. The ECM stiffness governs cell differentiation, proliferation, gene expression, and migration [19]. Thus, biomechanical environmental changes result in alterations in skin dynamics and skin appearance as well. As we age epidermis layer thins, the numbers of melanocytes and fibroblasts decrease, loss of collagen matrix and fragmentation of ECM become apparent, and fibroblasts can no longer attach the ECM resulting in reduced function and impairment of skin tissue integrity, leading to wrinkling formation [20,21]. On the other hand, subjecting the skin to external forces results in both cell-cell and cell-ECM interactions. When skin is projected to stress, actin filaments and collagen fibers align themselves with the load axis, and actin stress fibers are formed to promote nuclear translocation and transcription of genes connected to proliferation and differentiation. It is evident that the properties of skin, such as wound healing and age-associated changes, are governed by biomechanical cues, and thus studying dynamic forces is imperative [19,20].

Wounds are openings of the skin caused by burns, infections, illnesses, or physical damage impairing the normal functions of the dermal tissue. Wound healing involves a chain of cellular events which are strictly dependent on each other to successfully remodel the damaged skin tissue [21,22,23]. In order to understand and improve wound closure, numerous in-vitro wound assays were created, allowing non-invasive experimentation without the need for test animals/subjects [23,24,25].

Wound assays are commonly used to test the effects of active pharmaceutical ingredients, growth factors, chemokines, and cytokines enabling, accelerating, and improving cellular migration and proliferation in cell monolayers [22,23,24,25]. Each wounding method has its advantages and limitations. The most used in-vitro method for wound healing studies the “scratch assay,” in which a cultured cell monolayer will purposely be damaged via water/pressure jets, cell scrapers, pipette tips, lasers, heated stamps, and even electric currents [23,24,25,26,27,28]. Although simple and low-cost, a big disadvantage is the inhomogeneity and irregularity of the scratches, as well as the possible damage to the underlying matrix coating [28,29,30]. Stamping has similar disadvantages. However, it is less likely to damage any coating below the cell monolayer [25,31]. Thermo-mechanical systems which use heated stamps to create the wound area may often result in undefined gap sizes due to the heat transfer onto neighboring cells [25,31,32]. On the other hand, electric current, which leads to cellular detachment through induction, creates an irregular wound area and does not fully detach strong adhering cells such as fibroblasts or keratinocytes [31,32,33]. Lastly, focused laser beams are used for cell scraping through cell photo-ablation, which creates well-defined cell-free gaps [31,32,34]. However, it is costly and, therefore, not easily obtainable for smaller research groups.

For the comparability of different studies, a reproducible and standardized in-vitro method to visualize and quantify “wound” closure must be created. The different pathways those methods can activate must also be taken into consideration. In an optimal wound healing assay, gap sizes must be defined, the edges of the wound must be smooth, and the scarring of the cell monolayer must be completed with minimal to no harm to the neighboring cell.

In this study, we report the successful implementation of free-standing, ultra-thin silicon membranes, called the CellDrum, together with the novel steel-ball technique as a first step toward creating a reproducible in-vitro wounding model. Multivariate testing of parameters such as donor age and external cyclic biaxial stimulation, in contrast to standard culture flasks, is possible. We assessed, in particular, the initial wound perimeter as a quality measure of the wounding area and the gap-closure rate as wound closure. The data achieved here are based on common microscopic imaging techniques combined with the CellDrum technology to test pharmaceuticals, apply customizable mechanical stimuli, and examine the influence of the used stimuli on cellular functions.

## 2. Materials and Methods

### 2.1. Membrane Fabrication, Quality Management, and Surface Modifications

The PDMS elastomer kit Sylgard 184 from Dow Corning (Midland, MI, USA) was used as per the manufacturer’s directions. The PDMS base and the curing agent were mixed thoroughly in a 10:1 (*w*/*w*) ratio using positive displacement pipettes (Transferpettor, BRAND GMBH + CO KG, Baden-Wüttemberg, Wertheim, Germany) and centrifuged twice (Heraus Biofuge Primo, Thermofisher Scientific, Schwerte Germany) at 3200 rpm for approximately 2 min for degassing. PDMS membranes were fabricated with the FoW method [12]. Further information on PDMS membrane fabrication, membrane functionalization, and post-fabrication quality controls can be found elsewhere [13]. The membranes were taken for further quality assessments. The smooth, wrinkle and hole-free PDMS thin membranes were first autoclaved, and after sterilization, the thicknesses were measured by Filmetrics F20 (KLA Instruments, Milpitas, CA, USA) via spectral reflectance technique. Only the membranes between thicknesses 3.2–4.1 um were used for this experiment, and the rest were discarded to ensure the uniformity of membrane mechanical characteristics. A two-step wet-chemical functionalization protocol starting with the oxidation step pursued by silanization, was followed [13,35,36,37]. The oxidation solution was composed of H_2_O/H_2_O_2_ (30%) /HCl (37%) in a 5:1:1 volume ratio. After a 30 min wait time, membranes were washed, and the same step was repeated two more times without waiting time between washes. The silanization step was performed with a 2% solution of trimethoxy [2-(7-oxabicyclo [4.1.0]hept-3-yl)ethyl]silane in an Isopropanol/Water (19:1) mixture. The pH of the solution was regulated to 4.9–5.0 using acetic acid, and the mixture was kept on the membrane surface for 5 min. For biological functionalization, 1% fibronectin, acquired from bovine blood plasma (Sigma Aldrich, Taufkirchen, Germany) in MES buffer, was used, and CellDrums were kept in the refrigerator covered with phosphate buffer saline (PBS) solution until use.

### 2.2. Cell Culture

Normal human dermal fibroblasts from the abdomen region of female Caucasian adults of 28 and 88 years were obtained from Lonza, Germany (Lot No. 490824 and 664504, respectively). The cells were proliferated on standard cell culture flasks placed in a CO_2_ incubator at 37 °C. After reaching confluency at passage number 4, cells were detached according to the standard protocols, and 100,000 cells in 300 µL Dulbecco’s modified eagle medium (DMEM) supplemented with 10% fetal bovine serum (FBS) were seeded on the fibronectin-coated PDMS membranes (culture area 2.01 cm^2^). The CellDrums were incubated for a minimum of 24 h. The cells were checked for attachment and confluency under the Zeiss AxioVert inverted phase microscope.

### 2.3. Wound Model

**Figure 1:**
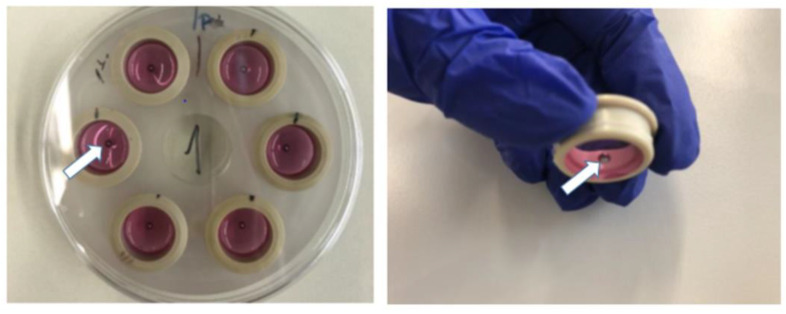
Representation of the 6-channel CellDrum holder used (**left**). Each individual CellDrum was filled with DMEM (pink-colored), and the steel balls were placed (white arrows) in the middle of the membranes (**right**).

Inspired by the stamping method, we decided to benefit from the mechanical properties of the CellDrum membrane and created a wound environment using steel balls. Stainless steel balls with a diameter of 2.5 mm (65.6 × 10^−3^ g per steel ball) were sterilized in an autoclave and placed in the middle of the membrane surface optically with sterilized tweezers. The CellDrums were incubated with the balls for 48 h. Medium change was performed as stated in the standard protocols. Due to the elasticity of the membrane, the weight of the steel balls led to increased pressure on top of the cell monolayer in the middle (Figure 1). The weight of the balls damaged the cell layer and forced the cells to migrate. The cell-free gap that was created on each membrane will be addressed as a “wound” in this paper.

### 2.4. Biaxial Mechanical Stimulation

**Figure 2:**
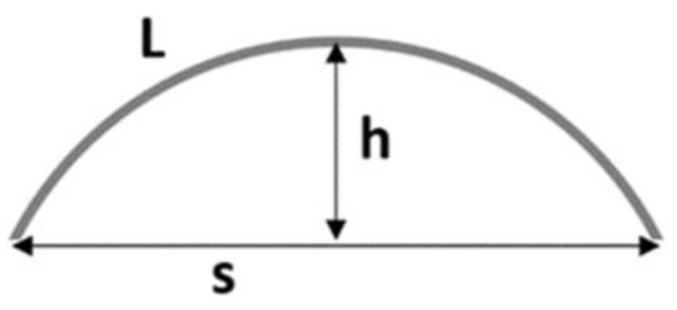
Graphical representation of the membrane deflection model. Value s, or the span of the circular arch, is the inner CellDrum applicator diameter. Value h is the deflection that can be measured by distance sensors.

The free-standing, flexible PDMS membrane of the CellDrums allows equibiaxial mechanical stimulation in contrast to the primarily used other mechanical stimulation methods such as uniaxial stretching. The PulSElect cell trainer for the CellDrum technology to apply biaxial stretching forces to the membranes is an innovation by Creutz [18]. It is composed of a loudspeaker, frequency generator, frequency distributor, signal visualization screen, and the CellDrum holder (slots for 12 CellDrums for simultaneous stimulation). The desired frequency and amplitude could be set with the frequency generator. The loudspeaker provides air pulse oscillations creating uniform, biaxial cyclic stretching. The most favorable models for in-vitro mechanical stimulation are generally below 5 Hz to avoid cell damage [38,39]. In other experiments completed in our laboratory, a cyclic stimulation of 1 Hz was shown to create a detectable ECM remodeling without harming the cell monolayer. Therefore, in this study, a frequency of 1 Hz and a peak-to-peak voltage (Vpp) of 10 was used. The strain amplitude was indirectly adjusted by setting the peak-to-peak voltage of the frequency generator to 10 V. Within the system used. This results in an average pressure amplitude of 7 Pa. With the help of a membrane deflection model, an approximate strain value of 5% was calculated (Figure 2).

Taking into consideration that the membrane is axisymmetric, the membrane strain can be calculated in a two-dimensional model. The general formula for calculating the strain is given below (Equation (1)).

(1) $$\varepsilon \left(Strain\right)=\frac{\Delta L}{L_{0}}$$

While $L_{0}$ is the default length, ΔL stands for the change of length. In the case of the CellDrum membrane, the length of the cross-sectional arch is taken as L. This arch is approximated to a circular arch with the formula (Equation (2)). The average downward deflection h of a CellDrum membrane when solely loaded with cell culture medium is obtained empirically. With Equation (2), one can calculate the corresponding length of L0.

(2) $$L=\frac{\arctan\left(\frac{2h}{s}\right)*\left(4h^{2}+s^{2}\right)*\frac{\pi }{180}}{2h}$$

Since the very low-pressure readings did not allow for a more precise calculation of the cellular strain, the main focus was laid on pulse frequency and duration. Fast calculation of cellular strain within the stimulation is the subject of ongoing trials.

In total, 40 CellDrum membranes were coated with dermal fibroblasts. By taking the two parameters, donor age and presence of mechanical training, into consideration, each 10 CellDrum (1 CellDrum = 1 sample) were then grouped together. For both ages, a control and training group was prepared, creating 4 separate groups.

48 h after wound creation, the steel balls were removed, the CellDrums were placed inside the airtight CellDrum holders, and the lid was secured. The cells were exposed to cyclic strain at 1 Hz for 30 min followed by a 2-h rest. This schedule was repeated three times, and between each session, cell media were renewed. On the second day of the training, the CellDrums were stimulated only one-time. This training protocol, although not published, was used in many experiments conducted in our laboratory. To determine if stretching causes cell detachment, samples were visualized with the phase microscope after every training session. Furthermore, in a previously unpublished study, live-dead cell imaging with fluorescent dyes was carried out using the same cell lines and after the same cyclic strain protocol used here to determine the number of unviable cells as a parameter of cell health. Out of 200,000 cells inoculated on the CellDrum membrane, approximately 514 dead cells were counted, corresponding to 0.7% of unviable cells [39]. Therefore, this training method was deemed to be suitable.

### 2.5. Wound Closure Visualization

In order to enhance the image quality and visibility, the cells were stained before mechanical stimulation using CellTracker**^®^** GreenCMFDA dye (Invitrogen ™, Karlsruhe, Germany). The staining was accomplished according to the manufacturer’s protocol; the vial was brought to room temperature; the lyophilized product was dissolved with 10.75 µL of Dimethyl sulfoxide (DMSO) to reach a final concentration of 10 mM. The stock solution was diluted with serum-free medium to a final working concentration of 0.5–25 µM. The working concentration that gives the best image quality and contrast was tested and found to be 5 uM. The cell media were aspirated from the CellDrums and replaced with pre-warmed CellTracker**^®^** dye. The CellDrums were incubated for 30 min in the CO_2_ incubator. The probe was aspirated and replaced with a culture medium.

### 2.6. Quantification of Gap Closure

The closure of the wounds created was monitored with the help of the CellTacker **^®^** dye and the fluorescence microscope KEYENCE Biozero. The custom holder was 3D printed to fit the CellDrum holder and locate the wound area. In order to make sure the same place is photographed each time, the coordinates of the inspected areas were noted down. The pictures were taken at the time points (1) initial before mechanical stimulation, (2) 7 h after three sessions of mechanical stimulation, (3) after 11 h, (4) 24 h after all stimulation sessions are completed, (5) 48 h, and finally at (6) 4 days.

A total of 240 images were gathered and analyzed using the internal software of the KEYENCE Biozero Fluorescence microscope. The wound area and the circumference of the gap were analyzed at the given time intervals above. In order to calculate the area, the polygon function was chosen, and the wound gap was manually outlined. The calculated value was then converted into square micrometers (μm^2^) and saved.

Gap closure was further calculated with the help of the Lundberg formula (Lundberg et al. 1992), given the parameters that were taken from every image (Formula 3) [33,40]. In the first formula, $Area_{i}$ defines the wound area at time i, a chosen time, and $Area_{0}$ is the initial scratch area. The gap-closure rate is then indirectly evaluated as the percentage of wound area at a chosen time point of interest.

(3) $$\mathrm{Gap}-\mathrm{closure}\;\mathrm{rate}(\%)=\frac{Area_{0}{-\;\mathrm{Area}}_{i}}{Area_{0}}\times 100$$

### 2.7. Statistical Analysis

Each experimental group was checked for normal distribution. As the results showed non-normality, it was decided to continue with a non-parametric test. To compare the wound perimeter of each sample between different time points, a one-tailed (right) Wilcoxon-Signed-Rank test was chosen.

## 3. Results and Discussion

### 3.1. CellDrum Membrane Production

Precision in the preparation and selection of the CellDrum membranes produced is crucial for the significance of the biological results. Only CellDrums that are as identical as possible in their parameters ensure identical mechanical stimulation of the cell cultures. Thus, we elaborated first on a standard laboratory procedure as described in Section 2.1 [13]. 48 CellDrum membranes were produced in total. After a visual pre-selection, further quality controls, and thickness measurements, 40 CellDrums were used for further tests. The used membrane thickness range can be seen in the table below (Table 1), including the average thickness value as well as the standard error of the mean. According to a previous study from our laboratory, membrane deflection and compliance values did not significantly differ in a thickness range of 3–4 µm, therefore, were not separately measured for the membranes used in this study [13].

**Figure 3:**
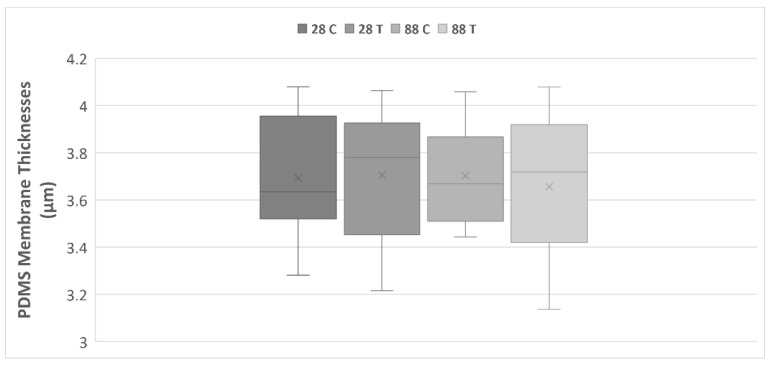
PDMS membrane thickness (µm) distribution for all experimental groups. The numbers 28 and 88 stand for the age of the donor which the dermal fibroblast cells were harvested from. C = Control Group and T = Training Group. The lower and upper fences are 25th and 75th percentiles, while the middle line represents the median, and the letter X stands for the sample average. The lower and upper whiskers show the minimum and maximum sample values, respectively.

Membrane production was completed successfully, and the grouping of the membranes was performed, taking membrane thicknesses into consideration, with the aim of having a similar distribution of the varying thicknesses, which can be seen in the following figure (Figure 3).

### 3.2. Trials with Different Methods of “Wound Generation” In-Vitro

Multiple measurements and experiments were conducted in order to decide on the most feasible and optimal wounding method on PDMS membranes. The most used approaches for wound modeling are scratch assay, vacuuming, and laser. For the scratch assay, the pointy end of common pipette tips (20–100 µL) was covered with parafilm to create a softer bulge at the tip, and the cell monolayers were carefully scratched. For the wound model created by the vacuum, the vacuum hand operator Vacuboy from Integra was used. The tip of the vacuum was slightly pressed on the PDMS membrane. The standard scratch assay was not feasible using PDMS membranes as it created irregular wound areas and occasional surface damage depending on the amount of force applied (such as rips or wrinkle-formation). Therefore, membranes that were damaged by the classic scratch assay method were not pictured. The suction pipette, Vacuboy, did not have a damaging effect on the membrane when used to create a cell-free gap, such as the above-mentioned scratch assay. However, the suction was not strong enough to detach the cells uniformly. This method was found not to be appropriate to use with strongly adhering fibroblasts. The steel-ball method, which was fundamentally inspired by the stamping method, was found to be the most viable method of all, creating no visible disturbances on the membrane surface itself in addition to the formation of a cell-free gap.

### 3.3. Gap Perimeter Standardization

**Figure 4:**
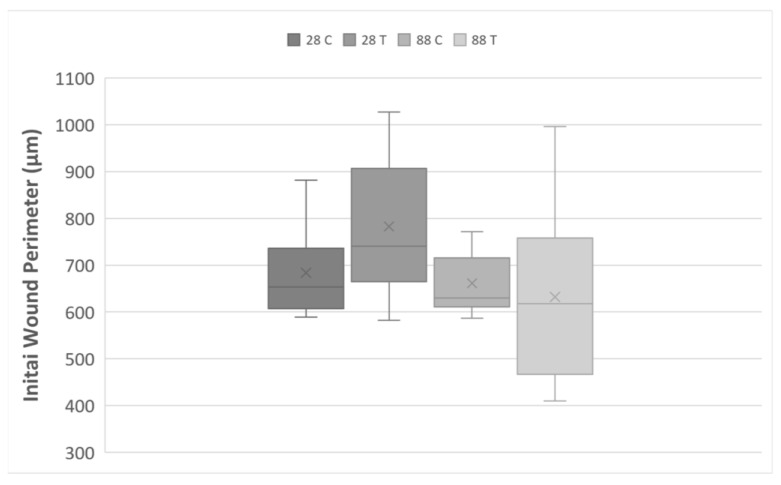
Boxplot of the initial wound perimeter created for all samples by steel balls (in µm). The lower and upper fences are 25th and 75th percentiles, while the middle line represents the median, and the letter X stands for the group average. The lower and upper whiskers show the minimum and maximum sample values, respectively. The numbers 28 and 88 stand for the age of the donor which the dermal fibroblast cells were harvested from. C = Control Group and T = Training Group.

To be able to assess the uniformity of the created wounding area by the steel ball technique, the initial wound perimeter was chosen as the investigated parameter. The measured initial wound perimeters in µm for all experimental groups can be found in Figure 4.

There were two outliers with perimeters 1112.13 µm and 1190 µm found in the 28-year-old control group and 28-year-old training group, respectively, and were removed in the upcoming calculations. These discrepancies resulted from the movement of the steel ball during the transportation of the samples. The free-standing, flexible surface of the CellDrum membrane allowed the relatively stable and stationary positioning of the steel balls. This was not possible on hard surfaces such as silicon or plastic culture flasks where accidental ball movement occurred, creating bigger gaps. The biggest and smallest wound areas recorded, excluding the outliers, were 1027.45 µm (28 T group) and 410 µm (88 T group), respectively. The 28-year-old control group had mean initial wound perimeter and SEM of 684.7 ± 35.1 µm; the 28-year-old training group 779.3 ± 47.7 µm; the 88-year-old control group 661.2 ± 19.4 µm; 88-year-old training group 632.1 ± 54.4 µm, with a mean wound perimeter of 687.02 µm. There was no significant difference between the initial wound perimeters of older and younger donors therefore, the discrepancies between the highest and the lowest values are attributed to the surface tension created by the cell monolayer on the PDMS surface being not always uniform as primary fibroblast cells from different donors tend to have varying capacity to form clusters which are heightened with serum deprivation [41].

### 3.4. Gap Perimeter Change as Function of Wound Closure

**Figure 5:**
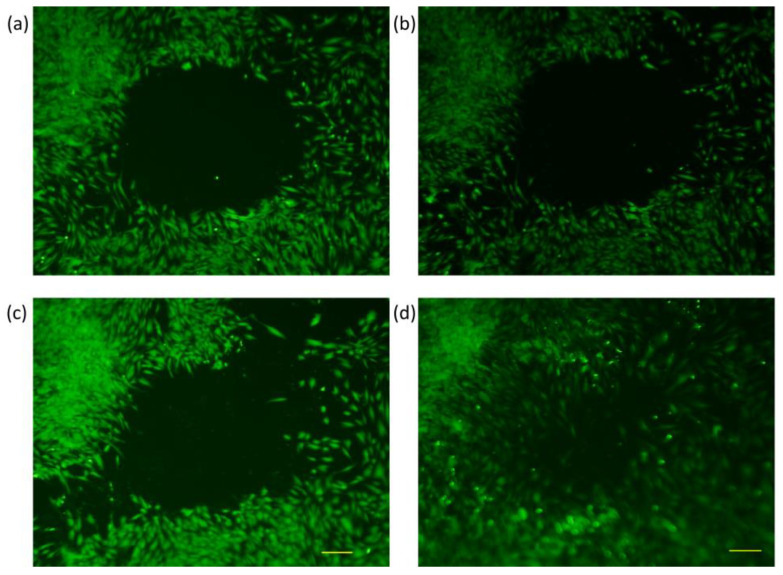
Fluorescence microscopy images of dermal fibroblasts from a 28-year-old donor at four different time frames (Control Group) (**a**) Initial, (**b**) 24 h, (**c**) 48 h, and (**d**) 4 days.

**Figure 6:**
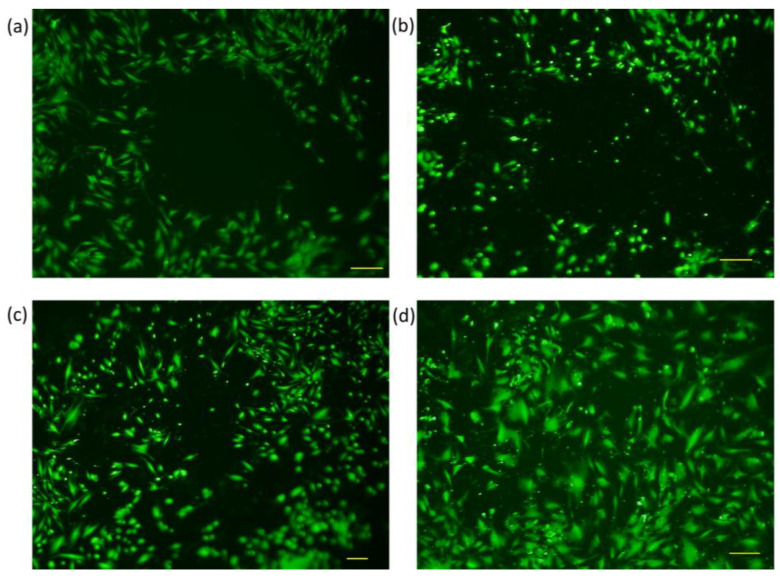
Fluorescence microscopy images of dermal fibroblasts from a 28-year-old donor at four different time frames (Training Group) (**a**) Initial, (**b**) 24 h, (**c**) 48 h, and (**d**) 4 days.

**Figure 7:**
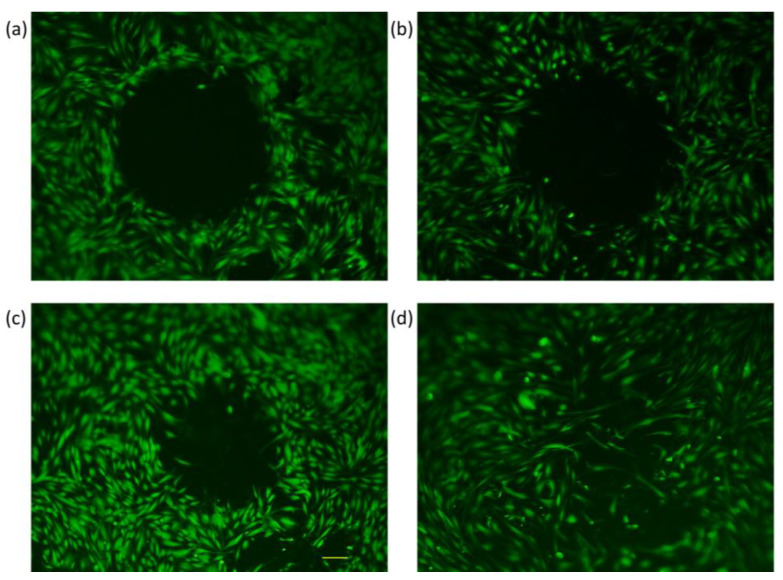
Fluorescence microscopy images of dermal fibroblasts from an 88-year-old donor at four different time frames (Control Group) (**a**) Initial, (**b**) 24 h, (**c**) 48 h, and (**d**) 4 days.

**Figure 8:**
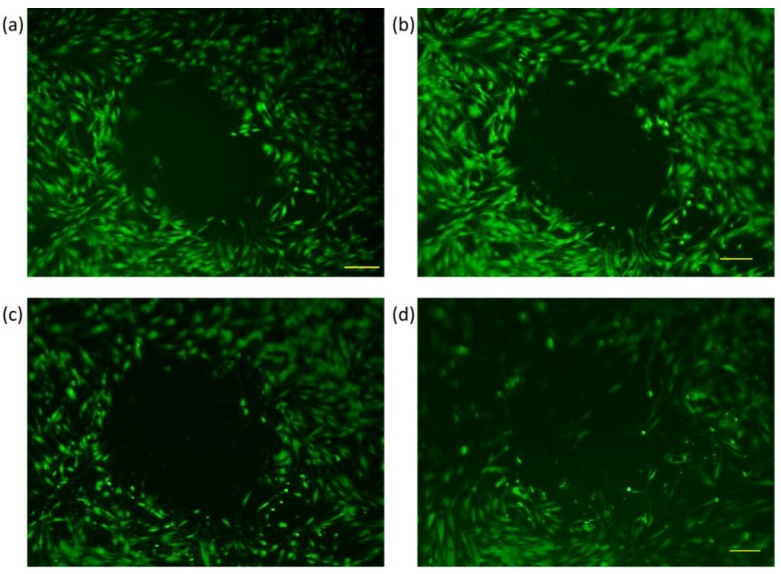
Fluorescence microscopy images of dermal fibroblasts from an 88-year-old donor at four different time frames (Training Group) (**a**) Initial, (**b**) 24 h, (**c**) 48 h, and (**d**) 4 days.

**Figure 9:**
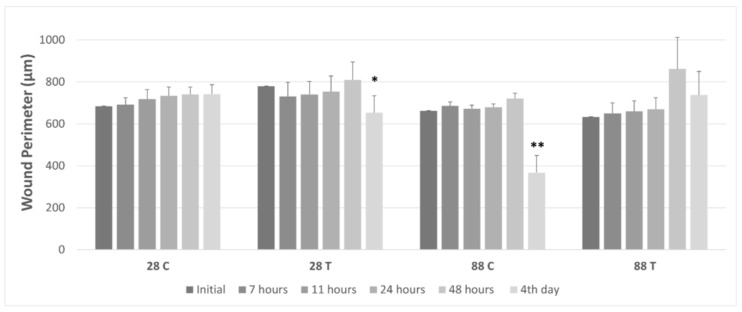
Cell training (1 Hz, 10 Vpp cyclic stretch) induced change of wound perimeter compared to non-trained cells (mean ± SEM) (* *p* < 0.05, significance compared to initial time point; ** *p* < 0.01, significance compared to initial time point). The numbers 28 and 88 stand for the age of the donor which the dermal fibroblast cells were harvested from. C = Control Group and T = Training Group.

Wound closure was tracked through fluorescence microscopy, wherein the perimeter of the gap created by the steel ball was monitored at different time intervals. The cell-free gap was outlined using the polygon function, and the perimeter size was calculated by the integrated microscope software. Pictures were taken at six different time frames, as explained under Section 2.6, and measurement values were recorded for every 10 samples in each group. An exemplary image of wound closure for every sample group can be seen below at four different time points (Figure 5, Figure 6, Figure 7 and Figure 8). The average numerical values for the gap perimeter can be seen in Figure 9 for all experimental groups.

As seen in Table 2 and Figure 5, Figure 6, Figure 7 and Figure 8, there is a seemingly contradicting pattern between the control and training group of dermal fibroblast cells of 88-year-old donors vs. 28-year-old ones overall. The wound perimeter seemed to get smaller after mechanical stimulation for the young donor, while 88-year-old donor cells showed an increase in wound perimeter with mechanical stimulation at the end of 4 days.

Table 2 and Figure 9 show the changes in the average wound perimeter for all samples. From the perimeter change values, the gap-closure rate of the samples was also calculated to assess and compare the effects of aging and the application of mechanical stimuli on wound healing. Numerical values for the gap-closure rate are shown in Table 3.

In the control group of the younger age donor samples, no significant change over the 4-day period was observed, with a gap-closure rate of −10%. Thus, it is considered to be negligible (Table 3). However, cell training seemed to have had a significant positive impact (*p* < 0.05) on wound closure after 4-day training (Table 2, Figure 6). The respective gap-closure rate was 33% collectively, showing a considerable improvement in wound closure in the presence of external mechanical stimuli. Training obviously improved wound healing in these experimental groups.

For the dermal fibroblasts taken from the 88-year-old donor, opposite reactions in the time dependencies of wound perimeter/gap-closure rate were measured. A highly significant decrease (*p* < 0.01) in the average perimeter values at the end of 4 days for the control group was indicated with a gap-closure rate of 55%, in contrast to the significant increase that is visible for the trained group with a gap-closure rate of −49% (Figure 7, Figure 8 and Figure 9).

As aforementioned, crucial functions of the skin are determined by the mechanical properties of the dermis integument, which regulates the skin’s elasticity and opposition to stretch. Dermal fibroblasts and their activities are profoundly affected by the surrounding extracellular matrix structure. Consequently, ECM stiffness governs cell differentiation, proliferation, gene expression, and migration, and thus stability and appearance of the skin/tissue [19]. Mechanotransduction of mechanical cues is fundamental for maintaining cell shape, directing migration, ECM organization, adapting cell morphology, and many more [42,43]. Therefore, biomechanical cues resulting from cyclic stretch used in this study cause both cell-cell and cell-ECM interactions. Dermal fibroblasts adapt and handle this stressful condition by rearranging F-actin structures together with collagen fibers parallel to the load axis forming stress fibers [19].

The external mechanical stimulation used in this study could have mimicked a wound contraction environment which naturally occurs during the in vivo wound healing process. This stimulatory effect could further explain the positive gap closure rate of the younger donor group. When a skin wound heals, fibroblasts produce tensile forces which allow the closing of a wound and a rapid total blockage of the wound with the help of the secreted collagen and other ECM components [44]. In a previous unpublished study from our laboratory, the gene expression of COL1A1 was upregulated by a fold change of 2.6 in 28-year-old donor fibroblast cells after mechanical stimulation. The same cell lines (28, 88-year-old donors) and mechanical stimulation parameters/system were used in this study. The mechanical stimulation used in this study could have simulated a late-phase injury environment in 28-year-old donor fibroblasts, which could have resulted in the increased fibroblast proliferation followed by higher collagen expression leading to better ECM remodeling, thus bigger and more significant wound closure in comparison to the control group [20,45,46,47]. Many past studies confirm the hypothesis of an increase in type I collagen (COL1A1) after exposure to uniaxial mechanical stretching in cardiac and ligament fibroblasts [20,45,46]. More recent and relatable studies using similar cyclic mechanical stresses showed accelerated proliferation and migration of dermal fibroblasts, adipose-derived stromal cells, mesenchymal stem cells, and chondrocytes in comparison to rigid cell culture [47,48,49,50].

A study from 2010 proved that the fibroblast stiffness between two experimental groups with two different ages showed a significant change, as the older cells presented 60% more rigidity in contrast to younger cells. The same study also reported a decrease in monomeric actin levels as a shift to filamentous actin. The stiffness change in the cell cytoskeleton due to aging can also further affect the cellular response to external stimuli, which can then lead to different gene expression profiles and that, in turn, could explain the contradicting responses of younger and older cells to the same external stress exposure [51,52]. In the same unpublished study above, a minor downregulation of COL1A1 by 34% in 88-year-old donor fibroblasts was seen. Age-related impairment of wound healing, together with decreased collagen expression, could explain the wound perimeter increase in the older donor control group by the 4-day mark (88 C).

When the skin ages, collagen production is reduced, and the collagen network, on which the fibroblasts are oriented is fragmented and damaged by the increased production of collagen-degrading enzymes [51,53]. When comparing the control groups with cells from young or old donors, we intuitively expected that the gaps in the cell cultures of young donors would close more quickly than those of older donors. However, our investigations did not confirm this. However, the biggest gap decrease and the highest gap closure rate (55%) were found for the 88-year-old control group (Table 3, Figure 9). Younger organisms are expected to have higher proliferation rates, better secretion of ECM proteins, and, overall, a faster wound closure rate as aging impairs the wound healing pathways and slows down most of the cellular functions [53,54]. However, a study from 2017, where three age groups (young, middle-aged, and old) were investigated, stated that the in-vivo aged skin fibroblasts do not show gene expression changes for genes COL1A1, TGFß1, CTGF, and MMP-I in-vitro. The gene expression of COL1A1 was found to be the lowest in the middle-aged group. The highest collagen content was found in males older than 40, whereas for women in a 65-year-old donor and some subjects younger than 30 years old, which contradicts the notion of decreased collagen expression in in-vivo aged skin. Kaisers et al. also showed that the changes in gene expression of in-vivo aged skin are not represented in short-term cultures [55]. Furthermore, in the unpublished study mentioned above, we have also seen a 75% increase in COL1A1 gene expression in the older age group compared to the younger group. The results we presented here are of cells that were used between passages 4–6 which was even shorter than what Kaisers et al. reported in their study. Cumulatively, these can clarify the unexpected decrease in the gap perimeter of the 88-year-old donor group (Figure 7 and Figure 8).

Overall, in this paper, we report the successful application of a novel technique to study wound closure on free-standing ultra-thin membranes. Cell lines from two different aged donors were used to test the effects of chronological donor age. Another tested parameter was the effect of external mechanical stimulation in the form of equibiaxial tensile stress. These two parameters were used to create four sample groups, each with 10 biological replicates, totaling up to 40 samples. Despite their optically visible load on the membrane in the form of membrane deflection, the steel balls were still able to move minimally on the cell monolayer. This led to minor discrepancies in the initial gap size. For future experiments, the use of a bigger range in donor age, as well as different mechanical stimulation parameters (frequency, amplitude, and/or stimulation time) would be beneficial to better understand the influences of above-mentioned changes. Bigger group sizes (>10) would further optimize the experimental setup by increasing the power of statistical analysis. Due to the short time period between cell seeding on the membrane and gap formation on the monolayer, performing a migration assay was not possible. In the upcoming experiments, cell starvation through the removal of the FBS 12 h prior to the placing of the steel balls, followed by the standard migration assay, will be conducted and compared with the herein-described method.

## 4. Conclusions

Various research has shown the need for standardized wound-healing assays to determine the rate of wound closure and measure the effects of numerous chemical stimuli. Our study demonstrates the use of a wound assay in which multiple parameters, such as age, and external mechanical stresses, could be tested simultaneously. The softer, flexible substrate allows for a better in vivo-like environment for the cell monolayer and makes it possible to test the effects of external mechanical stress. Varying intensities or frequencies of mechanical stimulation can also be tested for future studies of different cell types. Herein, we showed that the CellDrum system could be used to simulate a wound environment in-vitro creating uniform wound borders. The discrepancies in the age-related changes of dermal fibroblasts in this study can be explained by the fact that short-term cultures in which the cells are passaged no more than one to two times do not sufficiently mimic aged skin in vivo. This notion is supported by studies showing that, contrary to common belief, older individuals may have increased collagen accumulation, which has a positive effect on wound closure rates.

All in all, the easy monitoring of the changes in wound perimeters, along with the possibility to apply external mechanical stimuli in the form of equibiaxial pressure pulses, was possible thanks to the CellDrum technology. In the future, the CellDrum technology may further be used in tissue engineering or cosmetology through modeling or testing of new environments and chemical substances, as well as applying and measuring mechanical forces exerted on or by the cell monolayers. This paper can serve as a basis for an optimized and standardized wound assay using the novel pressure loading method on free-standing, ultra-thin, and flexible silicone membranes.

## Figures and Tables

**Table 1:** Descriptive statistics for membrane thickness values for all sample groups, with SEM (Standard Error of Mean). The numbers 28 and 88 stand for the age of the donor which the dermal fibroblast cells were harvested from. C = Control Group and T = Training Group.

Membrane Thickness in µm					
Experimental Groups		Average	SEM	Total Average	Total SEM
*n* = 10	28 T	3.70	0.09	3.69	0.04
	28 C	3.69	0.08		
	88 T	3.66	0.10		
	88 C	3.70	0.07		

**Table 2:** Average wound perimeter ± SEM in µm for dermal fibroblasts of 28- and 88-year-old donors. C= Control Group and T= Training Group. (* *p* < 0.05, significance compared to initial; ** *p* < 0.01, significance compared to initial).

Average Wound Perimeter in µm							
Experimental Groups		Initial	7 h	11 h	24 h	48 h	4 days
*n* = 10	28 C	683.7 ± 32.9	692.0 ± 33.3	717.7 ± 45.8	733.8 ± 41.6	740.1 ± 35.6	741.7 ± 44.7
	28 T	779.3 ± 45.2	730.6 ± 67.0	739.7 ± 63.0	753.4 ± 74.6	809.1 ± 85.3	652.8 ± 80.2 *
	88 C	661.2 ± 19.4	685.1 ± 18.7	672.3 ± 16.8	678.6 ± 16.6	720.2 ± 26.6	367.4 ± 81.6 **
	88 T	632.1 ± 54.4	649.6 ± 49.8	659.4 ± 49.9	669.2 ± 55.9	861.4 ± 151.2	737.2 ± 112.6

**Table 3:** Vectoral gap closure rates of all sample groups. The numbers 28 and 88 stand for the age of the donor which the dermal fibroblast cells were harvested from. C = Control Group and T = Training Group. Negative values indicate an increase in gap size and vice versa (* *p* < 0.05, significance compared to initial; ** *p* < 0.01, significance compared to initial).

Gap Closure Rate (%)				
Experimental Groups		24 h	48 h	4 days
*n* = 10	28 C	−10	−27	−10
	28 T	6	1	33 *
	88 C	−3	−17	55 **
	88 T	−14	−111	−49

## Data Availability

The data presented in this study are available on request from the first author.
